# IDENTIFYING CLASSES WITH DIFFERENT DISABILITY PROFILES AMONGST PATIENTS WITH PERSISTENT NECK PAIN

**DOI:** 10.2340/jrm.v58.46022

**Published:** 2026-09-02

**Authors:** Konsta KOIVUNEN, Mikhail SALTYCHEV, Juhani JUHOLA

**Affiliations:** 1 Clinical Division, Medical Faculty, University of Turku; 2 Department of Physical and Rehabilitation Medicine, Turku University Hospital and University of Turku, Turku, Finland

**Keywords:** disability evaluation, latent class analysis, neck pain, patient-reported outcome measures

## Abstract

**Objective:**

To identify latent classes with distinct disability profiles among patients with persistent neck pain using item-level responses from the Neck Disability Index, and to examine demographic, clinical, and lifestyle factors associated with class membership.

**Design:**

Cross-sectional survey-based study.

**Patients:**

962 patients with persistent neck pain.

**Methods:**

This survey-based study included 962 patients with persistent neck pain. Latent class analysis was performed using the 10 dichotomized items of the Neck Disability Index (NDI) as indicator variables. Demographic, clinical, and lifestyle characteristics were subsequently examined across the identified latent classes.

**Results:**

The mean age was 50 (SD 32.5) years and 647 (67%) were women. Three distinct classes were identified: class with no or mild disability (47% of the sample); class with moderate disability in reading, driving, and lifting but not in personal care and concentration (15% of the sample); and class with overall severe disability (37% of the sample). Lower educational level, obesity, physical inactivity, worse pain, and lower occupational status were associated with a higher probability of belonging to the severe disability class.

**Conclusion:**

Patients with persistent neck pain appear to exhibit distinct disability profiles rather than a single uniform pattern of functional limitation. Identifying these profiles may improve the characterization of patient heterogeneity and provide a basis for future research evaluating their clinical and prognostic relevance. Further studies are needed to determine whether these profiles are reproducible in other populations and whether they have implications for treatment planning and outcomes.

## LAY ABSTRACT

In patients suffering from persistent neck pain, the underlying causes of pain are diverse and so are the symptoms. Among these patients, not all may have the same limitations in their daily lives. However, it is not yet known whether some patient groups can be distinguished in which these limitations differ from average patients. In this study, a research method called latent class analysis was used to identify these groups among approximately a thousand patients suffering from persistent neck pain. Three distinguishable groups were found in which the severity of the patients’ overall limitations in functioning differed. Differences were also observed between the groups in which areas of everyday life were most likely to have problems. It was also found that some factors increased the likelihood of poorer functioning. Identifying these groups can help target rehabilitation and treatment interventions to the right patients.

Chronic neck pain affects over 200 million people globally as of 2020 (1). Patients with neck pain may often have difficulty performing everyday activities due to pain (2). It has been suggested that patients with neck pain are not a homogeneous group in terms of the spectrum of functional limitations (3, 4). Instead, specific domains of functional ability contribute differently to the disability experienced by different people. This heterogeneity has been studied previously, mostly in longitudinal data. Some previous studies have been able to identify larger groups of respondents in whom the change in disability level deviated from the average trend. These studies have generally used various statistical methods of trajectory analysis (5–7). For example, Yang et al. found 3 distinct groups of patients with cervical spondylosis based on pain severity: one-third were in the group with low pain, half were in the group with medium pain, and one-fifth in the group with severe pain (5).

Longitudinal trajectory analyses provide valuable information on how disability changes over time and how patients follow different recovery trajectories. However, they do not address whether patients with similar overall disability scores differ in the pattern of limitations across individual functional domains at a given time point. Cross-sectional latent class analysis (LCA) offers a complementary approach by identifying groups of patients with distinct disability profiles based on item-level responses (8). Such information may improve the understanding of heterogeneity among patients with persistent neck pain and provide hypotheses for future longitudinal investigations. The basic idea of LCA is to identify a few larger classes in a data set whose properties differ from each other.

While LCA has not previously been used to study disability profiles in patients with persistent neck pain, this method has been used to assess other similar situations, for example, the risk of neck pain in schoolchildren by age and sex (9), to map different musculoskeletal pain conditions (10, 11), to study disability profiles of psychiatric and diabetic patients (12), to assess the general characteristics of patients with persistent pain based on different questionnaires (13), to define groups with different patient phenotypes among patients with lumbar spinal stenosis (14), and to assign individuals to classes based on changes in function before and 2 years postoperatively after surgery for spinal deformity (15). Using the Oswestry Disability Index (ODI) as a main outcome measure, Rundell et al. have reported that 4 distinct patient groups were observed among patients with lumbar spinal stenosis: “high pain impact, low psychosocial features”, “mild pain impact, low psychosocial features”, “high pain impact, complex health needs”, and “acute, intermittent, moderate–severe leg pain with high pain impact” (14). Yang et al. have reported 4 distinct classes among patients operated on for spinal deformities: class with worsened disability particularly in lifting and pain intensity; class with stable disability in walking, standing, and sitting; class with mildly improved functioning in standing, social life, and employment; and class with substantial postoperative improvement especially in standing, social life, and work (15).

The most common scale to measure functional limitations caused by neck pain is the Neck Disability Index (NDI) (16, 17). The NDI consists of 10 questions concerning performance in different areas of daily life, providing an estimate of the total disability. Previous research on disability profiling has shown that different domains of functioning can affect the estimated overall level of disability in different ways for different people (18). That study used the NDI to assess patients who underwent cervical spine surgery. The patients had the greatest problems in coping with work and recreational activities, while there were fewer problems with self-care and concentration. Total NDI scores summarize disability but conceal potentially important differences in the pattern of functional limitations. Two patients may have exactly the same total NDI score while having completely different disability profiles. One may mainly have problems with driving and reading, whereas another may mainly have difficulties with sleeping and work. Total scores cannot distinguish between these patterns, but item-level latent class analysis can. Identifying patients with similar item-level disability profiles may provide clinically relevant information beyond the total score.

Based on previous evidence demonstrating heterogeneity in disability among patients with chronic musculoskeletal conditions, we hypothesized that patients with persistent neck pain would not constitute a homogeneous population but could be classified into distinct disability profiles according to their responses to individual NDI items. We further hypothesized that these profiles would differ in their demographic, clinical, and lifestyle characteristics.

It is not impossible to assume that using the NDI, which is a modification of the ODI, can also distinguish larger groups among neck pain patients in which the overall level of functional ability consists of more or less different domains of functioning. Such information may be of interest to both clinicians and researchers. Clinicians may use treatment measures that support the maintenance or improvement of the most severely impaired functional areas. Researchers may take the possible existence of these groups into account when studying neck pain patients in diverse settings. The aims of this study were (1) to identify latent classes with distinct disability profiles among patients with persistent neck pain based on item-level responses to the Neck Disability Index, and (2) to describe the demographic, clinical, and lifestyle characteristics of the identified classes.

## METHODS

This cross-sectional survey-based study included consecutive patients with neck pain referred to the outpatient Physical and Rehabilitation Medicine clinic of Turku University Hospital between April 2014 and February 2017. Referrals originated from primary care, occupational health services, private healthcare providers, and other specialized healthcare units. Although symptom duration was not recorded systematically, the waiting times at this outpatient clinic were such that the majority of patients were likely to have experienced neck pain for at least 2 months before their initial assessment. Before their physician appointment, patients completed a standardized questionnaire that included the Neck Disability Index (NDI), demographic information, pain intensity, perceived general health, work ability, and physical activity. The data did not contain a separate variable for symptom duration. We have clarified that patients were identified by completion of the NDI, which was completed only by patients reporting neck pain, and we have acknowledged this as a limitation. The study protocol was approved by the Ethics Committee of the Hospital District of Southwest Finland (ETMK 60/180/2012), and written informed consent was obtained from all participants prior to study enrolment.

Variables used to describe the latent classes were categorized to facilitate clinical interpretation of the class characteristics. Whenever possible, established clinical thresholds were used (e.g., obesity and severe pain); otherwise, pragmatic categorizations were applied. Categorization may reduce statistical information, but the purpose of these analyses was descriptive rather than inferential. Body mass index (BMI) was defined as weight divided by height squared and expressed in kg/m^2^. Patients were dichotomized based on BMI into groups of < 30 kg/m^2^ and ≥ 30 kg/m^2^. Educational status was dichotomized according to completion of general upper secondary school (high school: yes/no). Information on vocational education or higher educational attainment was not collected in sufficient detail to permit further classification. Occupational status was dichotomized as “managers and professionals” vs “technicians and manual workers”. The study database did not include a separate category for unemployed individuals. Consequently, occupational status was analysed using the categories available in the original questionnaire. Average pain intensity during the previous month was assessed using an 11-point numeric rating scale (NRS), ranging from 0 (“no pain”) to 10 (“worst imaginable pain”). Pain intensity was dichotomized as mild or moderate (< 7 points) vs severe (≥ 7 points), based on an established classification of NRS pain severity (19). Physical activity was assessed using a self-administered questionnaire in which participants reported their weekly duration of 4 categories of leisure-time physical activity: walking, brisk walking, light jogging, and vigorous running. The response options ranged from “none” to “4 hours or more per week”. Weekly physical activity was expressed as metabolic equivalent hours per week (MET-h/week) by multiplying the reported duration of each activity by its corresponding MET value (3.5, 5.0, 8.0, and 11.0 METs, respectively) according to the 2011 Compendium of Physical Activities, and summing the resulting values (20). The total MET-h/week was subsequently dichotomized as < 15 vs ≥ 15 MET-h/week. Age was defined in full years at the time of visiting a clinic. The sample was divided into 2 age groups of equal size based on median. Pain area was dichotomized as “only neck pain” vs “back and neck pain”. Biological sex was recorded as men or women. Marital status was dichotomized as single vs cohabiting (including married and cohabiting participants).

The Neck Disability Index (NDI) consists of 10 items assessing pain intensity, personal care, lifting, reading, headaches, concentration, work, driving, sleeping, and recreation. Each item is scored on a 6-point ordinal scale ranging from 0 (no limitation) to 5 (complete limitation) (16, 17). The total score is a percentage calculated by the sum of all answers divided by 50 (the maximum possible number of points) and multiplied by 100 as follows: Total score = (∑item scores/50) × 100. The equation is adjusted when the responses to 1 or more items are missing. A score of 0% represents the highest possible level of functioning and independence, whereas a score of 100% represents the lowest level of functioning with total dependence. Each response to each NDI item was dichotomized as 0–2 points “no or mild disability” vs 3–5 points “moderate or severe disability”.

### Statistical analysis

The descriptive statistics were reported as means and standard deviations (SD) or as frequencies and percentages, when appropriate. The main statistical method was LCA. The model included all 10 items of the NDI dichotomized as “no or mild disability” vs “moderate or severe disability”. The latent class model was based on the 10 dichotomized NDI items, which served as the indicator (manifest) variables defining the latent classes. Demographic, clinical, and lifestyle characteristics were not included in the latent class model itself but were analysed subsequently to describe the characteristics associated with each identified class. The LCA analysis began by determining how many latent classes could be distinguished in the sample based on the NDI responses. Models with 1 to 4 latent classes were evaluated. The 4-class model did not converge and was therefore not considered further. Model selection was based on several complementary criteria, including the Akaike Information Criterion (AIC), Bayesian Information Criterion (BIC), entropy, the Lo–Mendell–Rubin (LMR) adjusted likelihood-ratio test, model convergence, class sizes, and the clinical interpretability of the identified classes. Lower AIC and BIC values indicate better model fit, higher entropy indicates better classification accuracy, and a statistically significant LMR test suggests that a model with 1 additional class provides a better fit than the model with 1 fewer class. In addition to these statistical criteria, we considered whether the resulting classes were sufficiently large and clinically meaningful. The final model selection was based on a combination of statistical fit indices and clinical considerations. The 3-class model, where the AIC and BIC were closer to zero compared with the other model alternatives, was selected as the best one (Table SI). Following identification of the latent classes, demographic, clinical, and lifestyle characteristics were compared descriptively between the classes using cross-tabulations and Pearson’s χ^2^ tests. All tests were 2-sided, and a *p*-value < 0.05 was considered statistically significant.

Probability of each observed NDI item per latent class was reported along with 95% CIs. The list of indicators included several characteristics available in this sample: sex, marital status, educational status, occupational status, BMI, presence of neck or/and back pain, age, level of physical activity, and pain severity. All the indicators were dichotomized. The probability of each indicator for each class to define the characteristics of each class was analysed. All these probabilities were reported both numerically and graphically. No imputation of missing data was performed. Analyses were based on the available data for each variable; therefore, the number of observations may vary slightly between analyses because of missing values.

All analyses were conducted using Stata/IC Statistical Software: Release 19 (StataCorp LLP, College Station, TX, USA).

## RESULTS

This study included 962 patients, whose mean age was 50.0 (SD 32.5) years (Table I). Of the patients, 647 (67%) were women and 315 (33%) were men. The mean BMI was 27.0 (5.3) kg/m^2^.

**Table I:** Descriptive characteristics of sample (*n* = 962)^a^

Characteristic	*n* (%) or mean (SD)
Sex	
Men	315 (33%)
Women	647 (67%)
Marital status	
Single	232 (24%)
Cohabiting	730 (76%)
Educational status	
No high school	630 (68%)
High school	295 (32%)
Occupational status	
Managers and professionals	130 (14%)
Technicians and manual workers	832 (86%)
Current smoking	
No	678 (74%)
Yes	237 (26%)
Pain on average, points	7 (1.9)
Worst pain, points	8 (1.6)
General health	2 (1.1)
BMI, kg/m^2^	27 (5.3)
Alcohol consumption, g/week	35 (67.9)
WHODAS 2.0 total score, points 0–100	14 (9.6)
NDI total score, points 0–48	37 (17.5)
Age, years	50 (32.5)
Physical activity, MET/week	14 (13.6)

a The denominators vary slightly across variables because of missing data.

**Fig. 1:**
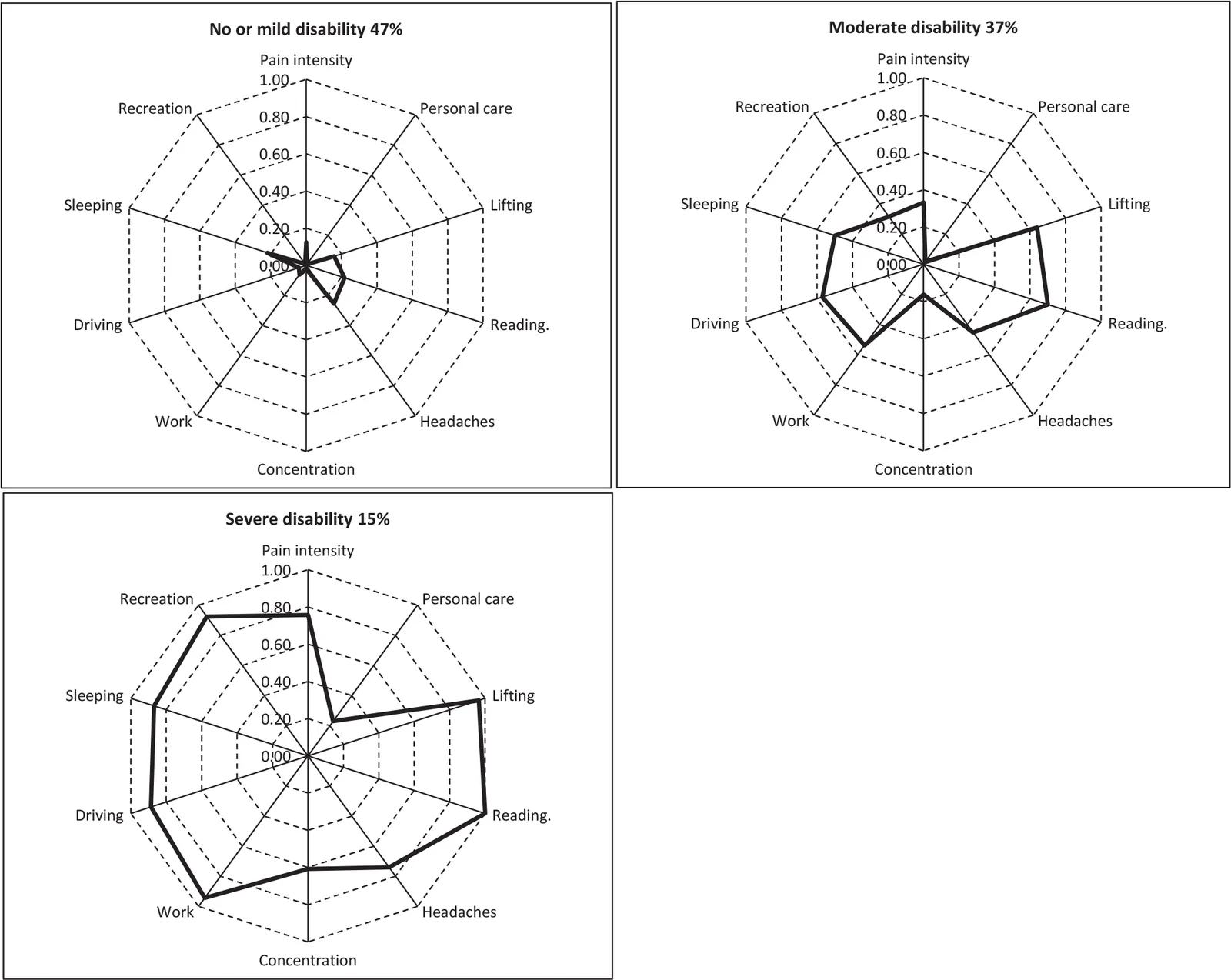
Distribution of probabilities of dichotomized items of Oswestry Disability Index for each latent class.

Three classes were identified (Table SII and Fig. 1). Fig. 1 shows the probability distribution of NDI items for each class. The probability of 1.0 represents the maximum probability of having severe disability in a specific domain.

### Class #1. No or mild disability

This class represented 47% of the sample (95% CI 41% to 53%). The probability of severe limitations in any of the NDI functional domains was 0.01 to 0.25. The lowest probabilities were seen for personal care (0.01, 95% CI 0.00 to 0.03), concentration (0.01, 95% CI 0.01 to 0.05), recreation (0.01, 95% CI 0.00 to 0.05), and driving (0.04, 95% CI 0.02 to 0.10), indicating that severe impairment in these domains was virtually non-existent.

### Class #2. Moderate disability

This class represented 15% of the sample (95% CI 12% to 19%). In this class, the probability of severe impairment in any of the NDI functional domains ranged from 0.02 to 0.70. The highest probability of severe limitations was seen (0.57, 95% CI 0.49 to 0.65). The lowest probabilities were seen in personal care (0.01, 95% CI 0.01 to 0.05) and concentration (0.16, 95% CI 0.12 to 0.22).

### Class #3. Severe disability

This class represented 37% of the sample (95% CI 32% to 43%). The probability of severe limitations in any of the NDI functional domains ranged from 0.23 to 0.94. The highest probabilities of severe limitations were in reading (1.00, 95% CI 0.00 to 1.00), work (0.94, 95% CI 0.85 to 0.98), recreation (0.92, 95% CI 0.82 to 0.97), lifting (0.96, 95% CI 0.90 to 0.99), driving (0.89, 95% CI 0.80 to 0.94), and sleep (0.87, 95% CI 0.77 to 0.93). The only domain unlikely to have severe limitations was personal care (0.23, 95% CI 0.16 to 0.32).

### Probabilities to be classified into a particular class based on some independent attributes

**Fig. 2:**
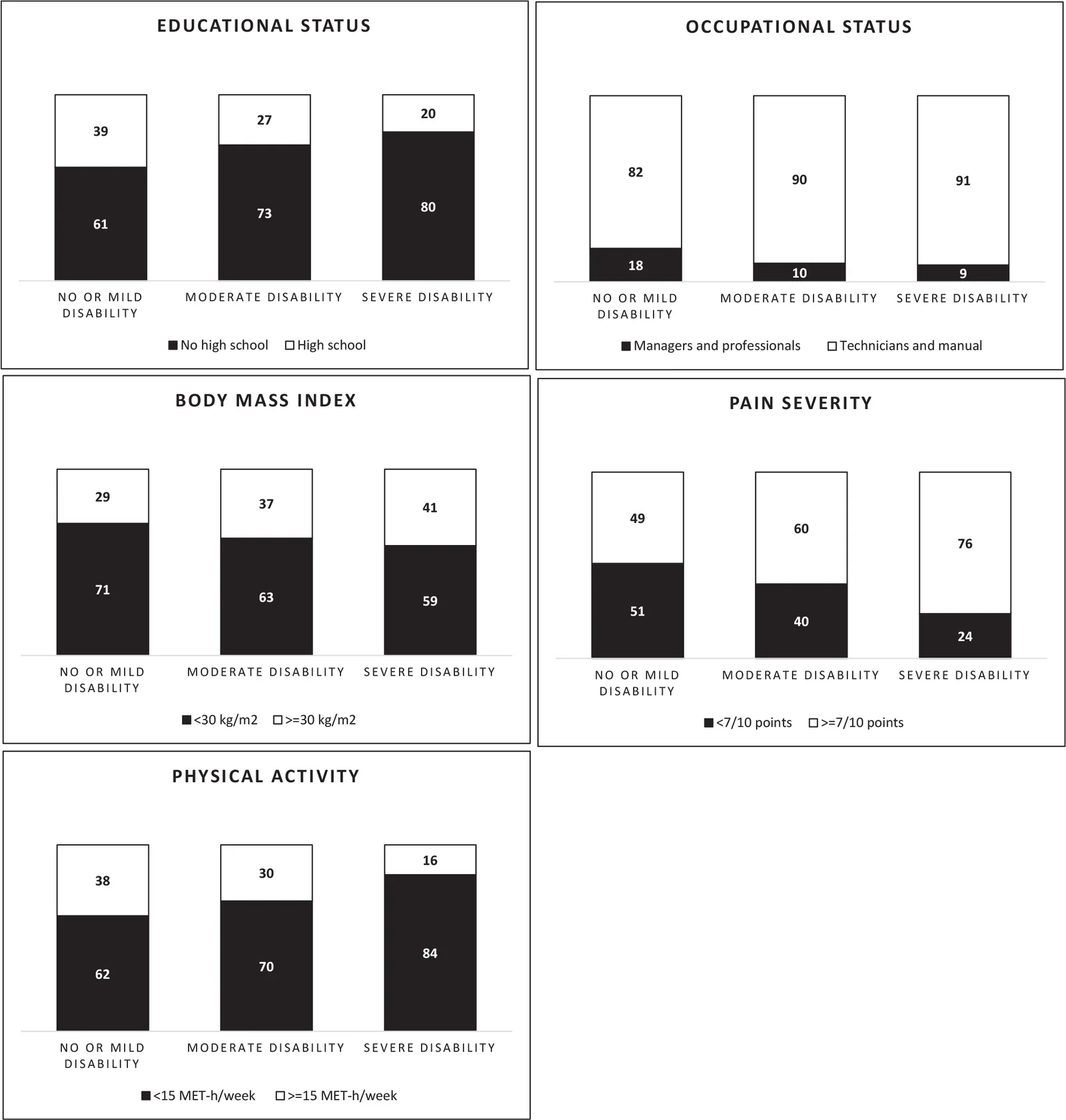
Probability of each indicator for each class to define the characteristics of each group. All *p*-values are < 0.001.

Fig. 2 provides a graphical summary of the principal differences between the latent classes, whereas the complete descriptive results, including all variables and corresponding *p*-values, are presented in Table SIII. The differences between the 3 groups were statistically significant for most attributes (Table SIII). When comparing class #1 (no or mild disability) with class #3 (severe disability), Fig. 2 shows that lower educational level (80% vs 61%), obesity (41% vs 29%), lower physical activity (84% vs 62%), worse pain level (76% vs 49%), and working as a technician or a manual worker (91% vs 82%) were associated with a higher probability of belonging to the severe disability class. Statistical significance was not achieved for sex, age, and marital status, indicating that these attributes were not clearly associated with belonging to the class with severe disability.

## DISCUSSION

In this cross-sectional study of 962 patients with persistent neck pain, 3 distinct classes were identified. Among all patients, 15% were classified as having moderate functional limitations, most likely in reading, driving, and lifting. The remainder of the sample was divided almost equally into 2 classes: in 1 class, patients had little or no functional limitations in any functional domain, while in the other, patients reported severe functional limitations in almost all functional domains. Lower educational level, obesity, physical inactivity, worse pain, and lower occupational status were associated with a higher probability of belonging to the severe disability class.

Disability is typically described using total scores from commonly used measures such as the NDI. However, when using the NDI total score, it is not possible to determine in which areas of everyday life a patient may particularly have challenges. Instead, it is often assumed that the population of interest is homogeneous and patients have the same level of limitations in all areas of functioning. This assumption is usually not true, especially when it comes to patients with persistent neck pain, whose symptoms and underlying causes vary widely (1, 21).

Despite the lack of previous studies using LCA for this purpose, these results can be indirectly compared to some previous knowledge related to other pain conditions (10, 11, 13, 22–24). As in this study, previous studies have observed heterogeneity in the spectrum of functional limitations in patients with persistent pain. Seven to 9 classes have been found among people with low back pain (24), 4 classes have been identified among people undergoing multimodal inpatient pain treatment (13), and 3 classes have been reported among people undergoing spinal deformity surgery (15).

Difficulties with reading, driving, and lifting seen in this study have also been among the most frequently reported problems in patients with persistent neck pain in previous studies (25). Patients with persistent neck pain may have problems with head stability and therefore may have proprioceptive deficits, which makes reading more difficult (26). Neck pain may also be associated with the fear of movement, which can limit the ability to lift heavy objects (27). Reduced cervical range of motion can make driving more difficult (28). Interestingly, pain intensity was not among significant functional limitations in any of the identified classes. This is in line with some previous studies, which have also reported that neck pain and disability may not be strongly correlated (29–31).

The role of lower educational level, obesity, physical inactivity, worse pain, and lower occupational status in being classified in a class with severe disability was in line with previous knowledge suggesting that these factors could be associated with worse functioning (32, 33). Contrary to some previous studies, female sex or older age did not increase the probability of belonging to classes with worse disability (34–37).

Previous epidemiological studies have reported varying prevalences of functionally disabling neck pain depending on the study population, case definition, and disability measure used. Neck pain is one of the leading causes of disability worldwide, affecting approximately 3–4% of the global population at any given time according to the Global Burden of Disease Study. However, these estimates describe the overall burden of neck pain rather than the heterogeneity of disability patterns among patients seeking specialist care. The present study complements these epidemiological findings by demonstrating that disability is not uniformly distributed across functional domains, even within a clinical population (38). Recent Global Burden of Disease estimates indicate that neck pain affected more than 200 million people worldwide in 2020 and remains one of the leading causes of years lived with disability. Our findings extend these epidemiological observations by suggesting that patients referred for specialist assessment comprise distinct disability profiles rather than a homogeneous clinical group (39). However, our findings should not be interpreted as prevalence estimates because the present study included patients referred to a tertiary outpatient rehabilitation clinic. Instead, the identified latent classes describe the heterogeneity of disability within this clinical population. Future population-based studies are needed to determine the prevalence and external validity of these disability profiles.

**Table II:** Probability of each observed item per latent class

Items	Probability	Standard error	95% CI	
Mild disability				
Pain intensity	0.12	0.02	0.09	0.17
Personal care	0.01	0.01	0.00	0.03
Lifting	0.16	0.02	0.12	0.21
Reading	0.22	0.03	0.17	0.28
Headaches	0.25	0.02	0.21	0.30
Concentration	0.02	0.01	0.01	0.05
Work	0.06	0.02	0.04	0.11
Driving	0.04	0.02	0.02	0.10
Sleeping	0.22	0.02	0.18	0.27
Recreation	0.01	0.01	0.00	0.05
Moderate disability				
Pain intensity	0.33	0.03	0.27	0.40
Personal care	0.02	0.01	0.01	0.05
Lifting	0.64	0.04	0.55	0.72
Reading	0.70	0.04	0.62	0.77
Headaches	0.45	0.03	0.39	0.52
Concentration	0.16	0.03	0.12	0.22
Work	0.54	0.05	0.45	0.62
Driving	0.57	0.04	0.49	0.65
Sleeping	0.50	0.04	0.43	0.57
Recreation	0.32	0.04	0.24	0.40
Severe disability				
Pain intensity	0.76	0.05	0.65	0.84
Personal care	0.23	0.04	0.16	0.32
Lifting	0.96	0.02	0.90	0.99
Reading	1.00	0.00	0.00	1.00
Headaches	0.74	0.05	0.64	0.82
Concentration	0.61	0.05	0.50	0.71
Work	0.94	0.03	0.85	0.98
Driving	0.89	0.04	0.80	0.94
Sleeping	0.87	0.04	0.77	0.93
Recreation	0.92	0.03	0.82	0.97

This study has several limitations. First, several descriptive variables, including body mass index and physical activity, were based on self-reported data and may therefore be subject to recall and social desirability bias. Second, the study population was recruited from a single tertiary outpatient Physical and Rehabilitation Medicine clinic in Finland. Consequently, the identified disability profiles may not be fully representative of patients with persistent neck pain in primary care or in other healthcare systems, and the generalizability of the findings should therefore be interpreted with caution. Because the study population comprised patients referred for specialist assessment, the findings may not be generalizable to community-dwelling individuals or patients managed exclusively in primary care. Finally, as already discussed, the cross-sectional design precludes conclusions regarding temporal or causal relationships between patient characteristics and disability profiles. Only a limited number of variables were available for analysis, and it is possible that some other factors increase the probability of worse disability. For example, there was no information on comorbidities available. Educational attainment was assessed only as completion of general upper secondary school, and more detailed information on vocational or higher education was unavailable. Consequently, educational status should be interpreted as a crude proxy rather than a comprehensive measure of educational attainment. Occupational status was recorded using a simplified classification, and unemployed individuals could not be identified separately. This may have resulted in some misclassification and should be considered when interpreting the associations involving occupational status. The present study was limited by the variables available in the original dataset. Information on psychological factors, such as depression, anxiety, pain catastrophizing, or fear of movement, as well as the frequency and duration of neck pain, was not available. These factors may contribute to the heterogeneity of disability profiles and should be incorporated into future studies. The present study also has several strengths. It included a relatively large sample of consecutive patients with persistent neck pain referred to a tertiary outpatient rehabilitation clinic, enhancing the clinical relevance of the findings. Rather than relying solely on the total NDI score, we analysed responses to individual NDI items, allowing a more detailed characterization of disability patterns. Furthermore, the use of latent class analysis provided a person-centred approach for identifying clinically interpretable disability profiles that may not be apparent when using conventional summary scores alone.

Aforementioned weaknesses lead to a suggestion that the analysis should be replicated in different settings. Also, other measures of functioning should be employed. The accuracy of LCA is directly proportional to the size of the data, so further studies with larger samples are needed. It would also be interesting to see whether the class division seen in this study prevails over time and to what extent it may change during the process of rehabilitation or treatment.

### Clinical and research implications

The identification of distinct disability profiles suggests that patients with persistent neck pain should not necessarily be regarded as a homogeneous group. Although the present cross-sectional findings do not support profile-specific treatment recommendations, they provide a framework for more detailed characterization of patients beyond the overall NDI score. Future longitudinal studies should examine whether these disability profiles differ in prognosis, treatment response, or healthcare utilization. If confirmed, such profiles may contribute to more individualized assessment and rehabilitation strategies. Our findings also highlight that patients with similar total NDI scores may have markedly different patterns of functional limitation. Considering item-level disability profiles in addition to the total score may therefore provide a more comprehensive understanding of patient functioning.

### Conclusions

Patients with persistent neck pain appear to exhibit distinct disability profiles rather than a single uniform pattern of functional limitation. Identifying these profiles may improve the characterization of patient heterogeneity and provide a basis for future research evaluating their clinical and prognostic relevance. Further studies are needed to determine whether these profiles are reproducible in other populations and whether they have implications for treatment planning and outcomes.

## Supplementary Material


