# Supplementary material for: IDENTIFYING CLASSES WITH DIFFERENT DISABILITY PROFILES AMONGST PATIENTS WITH PERSISTENT NECK PAIN
Source: J Rehabil Med. 2026 Sep 2;58:46022. doi: 10.2340/jrm.v58.46022 (PMC13548747; doi:10.2340/jrm.v58.46022)
Supplement: Supplementary file 1 [file JRM-58-46022-s1.pdf]

Table SI. Defining the number of latent classes. Retained model in bold.

	N	ll	Rank	AIC	BIC	Entropy	df	LMR	P>LMR
1-class	962	-5,070.86	10	10,161.71	10,210.40				
2-class	962	-4,367.22	21	8,776.44	8,878.69	0.7845	11	1,388.89	<0.001
**3-class [a]**	**962**	**-4,280.23**	**32**	**8,624.46**	**8,780.27**	**0.7117**	**11**	**171.71**	**<0.001**

[a] 4-class model did not converge. Therefore, a 3-class model was chosen for further analyses. AIC – Akaike information criterion; BIC – Bayesian information criterion; LMR – Lo–Mendell–Rubin-adjusted likelihood-ratio test statistic; Likelihood-ratio tests compare the given model versus the same model with one less latent class.

Table SII. Latent class marginal probabilities (sizes of latent classes as % of the sample).

Latent class/group	Size	Standard error	95% CI	
1	47%	3%	41%	53%
2	15%	2%	12%	19%
3	37%	3%	32%	43%

Table SIII. Distribution of probabilities to be classified into a particular class based on some independent characteristics.[A]

Indicator	No or mild disability	Moderate disability	Severe disability	Total	*p*-value
Sex					0.503
Men	151 (32%)	123 (35%)	41 (30%)	315 (33%)	
Women	318 (68%)	231 (65%)	98 (71%)	647 (67%)	
Total	469 (100%)	354 (100%)	139 (100%)	962 (100%)	
Marital status					0.531
Single	106 (23%)	89 (25%)	37 (27%)	232 (24%)	
Cohabiting	363 (77%)	265 (75%)	102 (73%)	730 (76%)	
Total	469 (100%)	354 (100%)	139 (100%)	962 (100%)	
Educational status					<0.001
No high school	277 (61%)	244 (73%)	109 (80%)	630 (68%)	
High school	176 (39%)	92 (27%)	27 (20%)	295 (32%)	
Total	453 (100%)	336 (100%)	136 (100%)	925 (100%)	
Occupational status					0.001
Managers and professionals	83 (18%)	35 (10%)	12 (9%)	130 (14%)	
Technicians and manual	386 (82%)	319 (90%)	127 (91%)	832 (86%)	
Total	469 (100%)	354 (100%)	139 (100%)	962 (100%)	
BMI					0.006
<30 kg/m^2	335 (71%)	224 (63%)	82 (59%)	641 (67%)	
>=30 kg/m^2	134 (29%)	130 (37%)	57 (41%)	321 (33%)	
Total	469 (100%)	354 (100%)	139 (100%)	962 (100%)	
Pain severity					<0.001
<7/10 points	238 (51%)	140 (40%)	33 (24%)	411 (43%)	
>=7/10 points	231 (49%)	214 (60%)	106 (76%)	551 (57%)	
Total	469 (100%)	354 (100%)	139 (100%)	962 (100%)	
Physical activity					<0.001
<15 MET-h/week	291 (62%)	247 (70%)	117 (84%)	655 (68%)	
>=15 MET-h/week	178 (38%)	107 (30%)	22 (16%)	307 (32%)	
Total	469 (100%)	354 (100%)	139 (100%)	962 (100%)	

Age group					0.328
Mean 36 years	245 (52%)	167 (47%)	68 (49%)	480 (50%)	
Mean 60 years	223 (48%)	187 (53%)	71 (51%)	481 (50%)	
Total	468 (100%)	354 (100%)	139 (100%)	961 (100%)	

[A]The denominators vary slightly across variables because of missing data.
